# Distance, weather, and forage conditions drive timing of autumn migration in female mule deer

**DOI:** 10.1186/s40462-025-00540-x

**Published:** 2025-02-25

**Authors:** Colby B. Anton, Nicholas J. DeCesare, Collin J. Peterson

**Affiliations:** 1 Montana Cooperative Wildlife Research Unit, University of Montana, Missoula, MT 59812 USA; 2 Present Address: Montana Fish, Wildlife and Parks, Missoula, MT 59804 USA; 3 Montana Fish, Wildlife and Parks, Missoula, MT 59804 USA; 4 Montana Fish, Wildlife and Parks, Dillon, MT 59725 USA

**Keywords:** Climate, Hunting, Migration, NDVI, *Odocoileus hemionus*, Precipitation, Snow water equivalent, Temperature

## Abstract

**Background:**

Seasonal migration is a behavioral strategy that animals evolved to exploit seasonally changing resources. Ungulates in northern temperate landscapes often seasonally migrate between low-elevation winter ranges and higher-elevation summer ranges, allowing individuals to exploit a diversity of forage resources during summer while avoiding extreme conditions during winter. In autumn, the timing of this behavior often overlaps with hunting seasons for managed ungulate populations. Migration presents challenges for managing ungulates when the timing of autumn migrations varies across years and migrations cross management jurisdictions.

**Methods:**

We evaluated the spatial and temporal patterns of autumn migration using GPS collar data collected during 2017–2019 from 68 female mule deer (*Odocoileus hemionus*) that migrated seasonally within three study areas in northwest Montana. We related the timing of autumn migration to environmental variables including precipitation, snow depth and density, temperature, plant phenology, migration distance, and estimates of relative hunting intensity. We summarized variables across multiple temporal scales (2-day, and 1 week) to identify possible lagged or cumulative effects of conditions on mule deer behavior. We incorporated these variables into a time-to-event modeling framework to estimate their relative impacts on the timing of initiation of autumn migration.

**Results:**

The collective annual space use of deer in each study area spanned up to 9 hunting districts, and individual deer used an average of 2.1, 2.8, and 2.0 hunting districts per year (range 1–4) in the Cabinet-Fisher, Rocky Mountain Front, and Whitefish study areas, respectively. Furthermore, the return of deer to winter ranges occurred over a 3-month timeframe spanning archery, rifle, and closed hunting periods. While some deer returned to winter range relatively early during archery season in September, others remained in summer range into December, after the general rifle season concluded. Declines in daily minimum temperatures and increased weekly precipitation provided the strongest cues for mule deer to begin their autumn migration. Mule deer with longer migration distances were more likely to initiate their migration sooner, and declining forage conditions also showed a modest effect on timing. Mule deer migrations occurred during times of lower hunting activity prior to its peak during rifle season.

**Conclusions:**

Our study demonstrates changing weather conditions were the primary driver of the initiation of autumn migration for mule deer. Given most migrations spanned more than one hunting district, the boundaries of management units were mismatched with the scale of ecological processes, implying that management actions in certain districts may have unintended consequences for populations in nearby districts.

**Supplementary Information:**

The online version contains supplementary material available at 10.1186/s40462-025-00540-x.

## Background

Seasonal migration is a complex behavioral strategy common across many taxa where individuals seek to exploit or avoid seasonally changing resources through relatively long-distance movements [1]. These highly directed movements often cross administrative and ecological boundaries and can have population-level influences [2]. Ungulates in northern temperate landscapes predictably migrate seasonally between low-elevation winter ranges and higher-elevation summer ranges [3–5]. Important to managing populations that display such migration patterns is understanding which cues are used to begin their movement to the next seasonal range. Thus far, spring migration has been examined with its timing attributed to several factors including tracking phenological green-up, commonly referred to as surfing the “green wave” [6]. This phenomenon leads to high synchrony in spring migration timing within populations.

In contrast to spring migration, snow depth and temperature changes have been identified as the main drivers of autumn migration timing [5, 7], but the senescence and declining availability of forage [8] and shifts in mortality risk may also play roles in cueing autumn migration [9]. Weather conditions often vary spatially, and animals can respond to these dissimilarities on differing temporal scales to avoid unsatisfactory conditions. As a result, spatial variation in environmental factors can strongly influence autumn migration timing [10]. For ungulates like mule deer (*Odocoileus hemionus*), deep snow can pose particularly difficult conditions leading to more energetically costly travel and partial or complete burial of higher quality forage resources [11, 12]. Moreover, increasing snow depth in autumn coincides with subsequent drops in ambient temperatures thereby compounding the energetic losses of travel through snow with the increased costs associated with thermoregulation [7]. Archery and rifle hunting can also induce heterogeneous spatiotemporal patterns of mortality risk, and lead to behavioral alterations for some animals [8, 13]. This period of increased human-related risk typically coincides with ungulate autumn migration, leading to the possibility that animals could alter their migration timing in response to hunting-induced risk or refugia [8, 9]. As migratory behaviors decline worldwide [14], autumnal climate patterns change [15], and hunting seasons remain an important tool for ungulate management, it is important to quantify the relative impacts of these variables as triggers of autumn migration.

Seasonally migratory game species often cross multiple administrative boundaries like hunting districts on an annual basis, posing challenges to wildlife managers. Migration across administrative boundaries reflects a mismatch between the spatial scale of management units and the scale at which ecological processes influencing populations occur [16]. These features may decouple management actions, like sex-specific harvest regulations, quotas, or season dates, from their intended effects on populations. Given that autumn migration and managed hunting seasons often overlap, at least partially, in time, individuals may be exposed to different harvest regulations or pressure as they traverse administrative boundaries [17]. Understanding how autumn migrations occur relative to the timing and spatial arrangement of harvest opportunity can guide effective wildlife management at ecologically relevant scales.

We studied autumn migration for 3 partially migratory populations of mule deer, where some animals seasonally migrated while others remain in the same range throughout the year [18], in Montana, with attention to: (a) the spatial and temporal overlap of migration with administrate boundaries such as hunting districts, and (b) factors driving the initiation of autumn migration for mule deer. Mule deer are an important game species in Montana, with an average of 152,000 hunters afield each autumn [19]. We developed five hypotheses to explain the timing of animals leaving summer range in this system. First, we hypothesized that snow depth could reduce access to forage and increase energetic expenditure, such that mule deer would initiate migration in response to snow accumulation [6]. Second, we hypothesized minimum temperatures could provide a separate cue such that temperature would be negatively correlated to the probability to initiating migration. Third, plant forage can shape ungulate landscape use and seasonal migration [20], guiding movements to track higher forage biomass as it changes through phenological stages [6]. Mirroring results that spring migration tracked plant phenology, we predicted migrations would start when plants had senesced on summer range and the availability of forage relative to winter range had decreased. Fourth, human hunting activity can alter habitat selection of ungulates and induce movements away from disturbances [21]. We predicted a positive correlation between hunting activity and probability of initiating migration. Lastly, we hypothesized that individuals would vary in their total distance travelled during migration. We predicted that mule deer with longer distance migrations would begin autumn movements earlier [8].

## Methods

### Study area

**Fig. 1:**
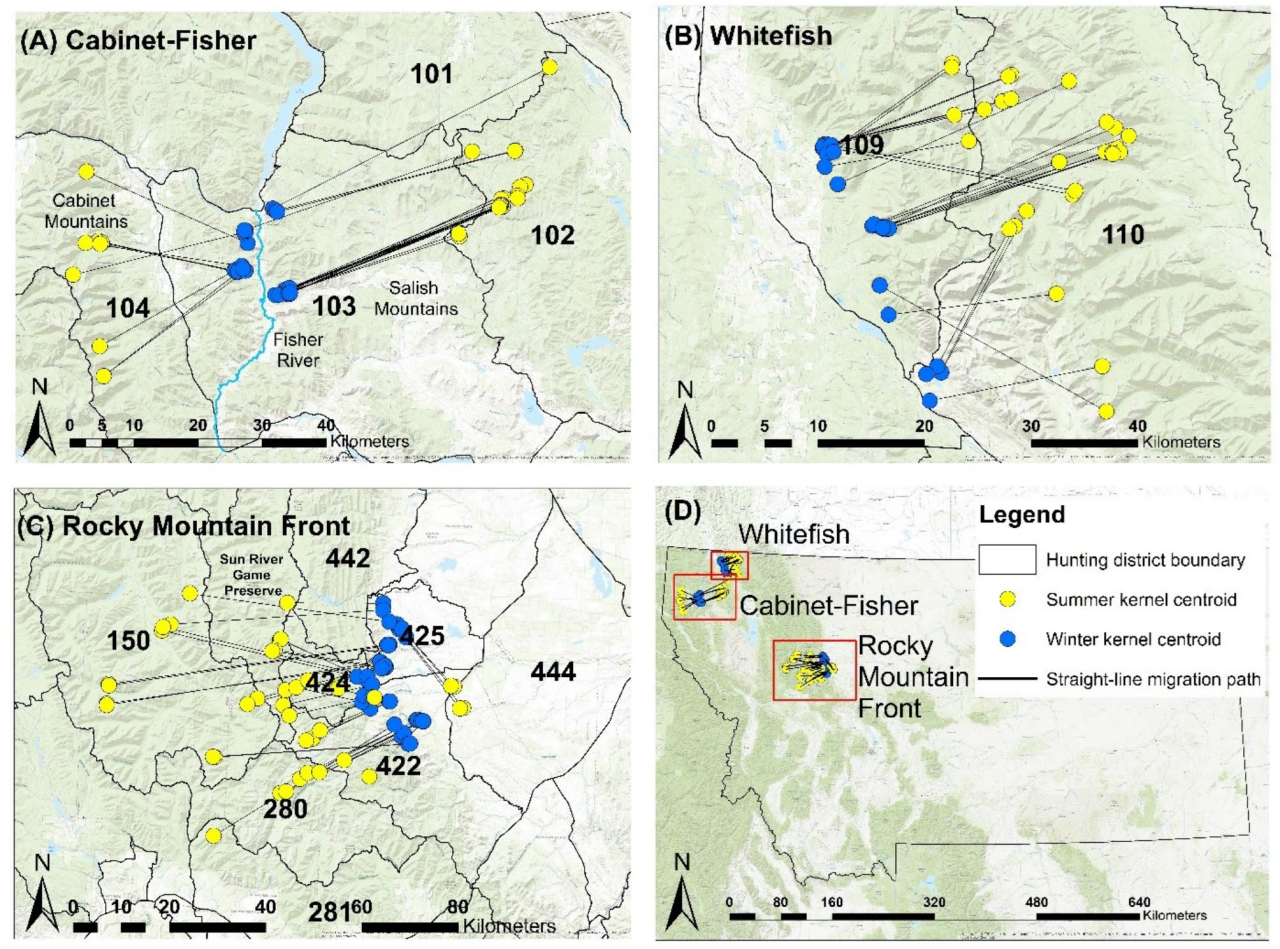
Map displaying mule deer summer (yellow) and winter (blue) home range centroids in each of the three study areas in northwest Montana: (**A**) Cabinet-Fisher, (**B**) Whitefish, and (**C**) Rocky Mountain Front. Black lines connect each summer and winter home range for a mule deer during an individual year

**Fig. 2:**
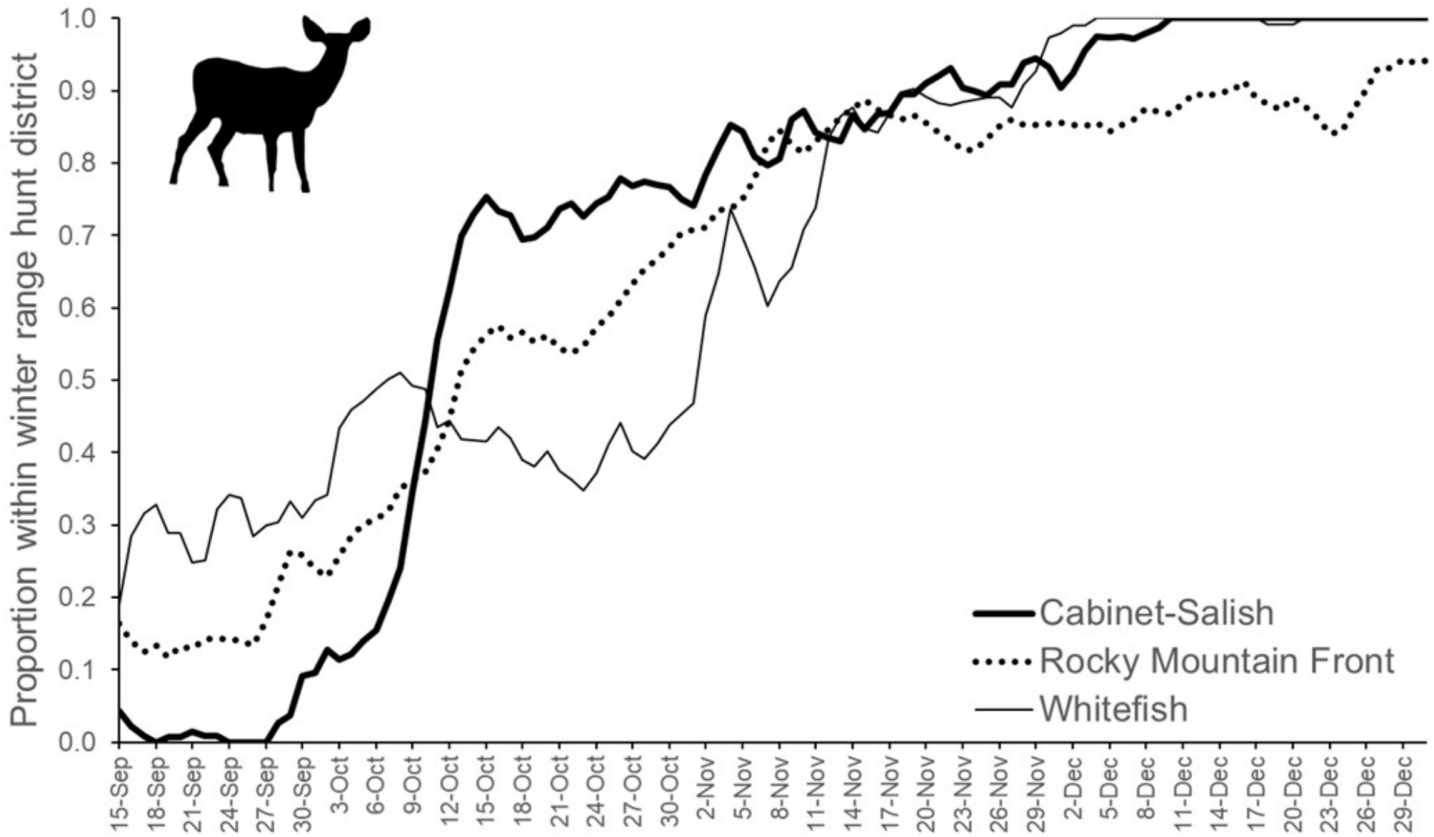
Proportion of migratory female mule deer that had returned to the hunting district encompassing their winter capture locations, using a 3-day average to smooth lines, averaged across years, and within 3 study areas of western Montana, 2017–2020

We studied mule deer in three study areas in northwest Montana (Fig. 1). The Cabinet-Fisher study area straddled the northern terminus of the Fisher River with elevations from 630 to 1760 m. Mule deer in the Cabinet-Fisher are partially migratory, including both eastward summer migrations into the Salish Mountains and westward migrations to the Cabinet Mountains. The area primarily comprises conifer forest with open shrub and grassland on south facing slopes. The study area also includes a substantial footprint of timber harvests and wildland fires. Autumn temperatures ranged from − 16 to 36° C (Figure S4) and average daily snow depth for each winter during the study period (2017–2020) ranged from 1.58 to 4.52 cm. Except for the low snowfall observed in 2020, snow depth was comparable to the average, 4.43 cm, from 2004 to 2020 (Fig. 2 and Figure S1). The annual range of Cabinet-Fisher mule deer overlapped that of four other native ungulate species, including moose (*Alces alces*), elk (*Cervus canadensis*), mountain goat (*Oreamnos americanus*), white-tailed deer (*Odocoileus virginianus*). Predators in this area include mountain lions (*Puma concolor*), coyotes (*Canis latrans*), wolves (*Canis lupus*), black bears (*Ursus americanus*), and grizzly bears (*U. arctos*).

The Rocky Mountain Front study area was a 1,357 km^2^ area on the western terminus of prairie habitat as the landscape transitioned into mountainous terrain (Fig. 1). The Sun River bounded the northern end and the Dearborn River the southern end of the study area. Elevation ranged from 1280 to 2510 m with much of the lower elevations dominated by montane grasslands. Deciduous shrubland interrupted larger swaths of grasslands as elevations increased into the foothills of the Rocky Mountains and dry-mesic conifer forests became the dominant land cover type. This area included large extents of privately-owned agricultural lands abutting public National Forests. The herd is partially migratory with most spring migrations leading animals westward into the Bob Marshall Wilderness. During the winters of this study, temperatures ranged from − 22 to 33° C (Figure S5) and snow depth varied from 3.65 to 10.4 cm. Except for 2020, average snow depths were higher than the average of 3.55 cm from 2004 to 2020 (Fig. 2 and Figure S2). The annual range of Rocky Mountain Front mule deer overlapped a similar suite of ungulate and predator species compared to Cabinet-Fisher, with the addition of pronghorn antelope (*Antilocapra americana*) and bighorn sheep (*Ovis canadensis*).

The Whitefish study area was a 794 km^2^ area on the west side of the Whitefish Mountain range, where elevations ranged from 850 to 2280 m (Fig. 1). Whitefish was dominated by wet and mesic conifer forests mixed with regenerating forest patches resulting from fire and timber harvest. The west end of Whitefish included human development and a two-lane highway. The Whitefish herd was also partially migratory with most animals migrating to summer ranges to the east into the Whitefish and some animals migrating farther to the northeast across the Canadian border. Temperatures ranged from − 16 to 36° C (Figure S6) and yearly snow depths averaged 19.59–28.81 cm. Snow depths during 2017, 2018, and 2020 were greater than the average (Figure S3), 20.9 cm from 2004 to 2020. The annual range of Whitefish mule deer overlapped a similar suite of ungulate and predator species compared to Rocky Mountain Front, with the exceptions of pronghorn and mountain goats.

Mule deer hunting regulations during the study period in these portions of Montana typically allowed for general, over-the-counter, licensed hunting of antlered male mule deer in most districts during a 6-week archery season (early September to mid-October) and a 5-week rifle season (late October to late November). Exceptions to this paradigm for general license mule deer hunting were many, including a shortened season length for antlered males in one hunting district (HD) (109), an early September 15th transition from archery to rifle season in some backcountry HDs (150, 280), additional general license opportunity for antlerless mule deer harvest on private lands in some HDs (422, 442) and on all lands in other HDs (444, 445), and one area completely closed to all hunting (Sun River Game Preserve). Concurrent general license hunting of elk and white-tailed deer occurs generally in the same districts and during the same seasons. Timber harvest occurred at varying levels across all three study areas but was largely finished for the season when mule deer migrations took place. In addition, no natural-gas extraction occurs in the region.

### Deer migration

Global positioning system (GPS) telemetry collars were deployed on 134 mule deer during December to March 2017–2019 (41 in Cabinet-Fisher, 49 in Rocky Mountain Front, and 44 in Whitefish) using helicopter gunning, clover traps, and ground darting. GPS collars were programmed to collect GPS locations every 13 h. Institutional Animal Care and Use Committees at the University of Montana and MFWP approved capture protocols (Animal Use Protocols 001-17CBWB-011017 and FWP03-2016), which align with standards established by the American Society of Mammalogists for research on wild animals [22]. We identified migration events using the Migration Mapper application [23], which uses net squared displacement curves to aid in the classification of migration timing [24]. We defined the first day of autumn migration for each deer and year as the first day the animal displayed directed movement out of their summer home range towards their winter home range. Movements were identified as a migration if they met the following criteria: (1) After leaving summer range, the mule deer never returned, (2) After leaving summer range, the mule deer moved to winter range. Migrations often included layovers where mule deer paused in local areas for 1–2 days before continuing migrations to winter range. The end of migration was determined by the first GPS location within the deer’s winter home range, regardless of the behavior of movements following this point in time. After identifying autumn migration events, we excluded outlier animal-years (9 deer migrations) that left summer range before July 31 or after December 31, and we used a subset of GPS data for each mule deer that began on August 26th of each year and ended on each animal’s first day of migration. We chose August 26th to include at least 2 weeks of location data prior to their initiation of migration for all animals. Lastly, for each deer we also calculated migration distances in km and the duration of migrations, measured in days, including layovers.

Prior to statistical assessment of the factors driving the initiation of autumn migrations, we first conducted some descriptive summaries of how the space use of migratory deer related to administrate boundaries such as hunting districts. Within each study area, we estimated the number of hunting districts spanned by migratory deer at the population and individual level. We then grouped deer according to HD within which they were captured during winter and summarized the daily proportion of deer within each hunting district as the autumn season progressed. Specifically, we summarized the proportion of GPS locations within each HD per day of the hunting season, beginning with the first day of archery season and ending on the last day of general rifle season each year. The goal of these descriptive summaries was to visualize how migration patterns affected the distribution of deer across different administrative boundaries throughout the hunting season.

### Environmental covariates

We summarized daily climatic and anthropogenic variables across multiple time scales for each deer location. Recognizing that mule deer may respond to both instantaneous and cumulative changes in the environment (e.g., a single snowstorm vs. a gradual accumulation of snow), we assessed conditions experienced by each deer according to weekly, 2-day and daily time scales. We used daily 1-km resolution raster data from the Daymet database to characterize spatiotemporal variation in precipitation, snow water equivalence (SWE), and minimum temperature [25]. SWE captures both depth and density of snow, both factors that could impede efficient travel by deer. In addition to daily measurements, we also calculated moving window 2-day and weekly averages for each climate variable. Additionally, we subtracted weekly averages from daily values as a metric of daily change.

To capture changes in plant phenology, we used both daily 250-m resolution normalized difference vegetation index (NDVI) from the MODIS MOD09GQ database and annual summary metrics calculated for each pixel from sequences of NDVI data, including the day of year associated with the end of measurable photosynthesis and the NDVI value reached on that day, available from the USGS Remote Sensing Phenology database [26]. We then subtracted the end of season day and NDVI values from daily MODIS data at each location to estimate metrics of the status of photosynthetic decay (NDVI_senescence_) and time (NDVI_season day_) relative to the end of the season. We assumed decreasing NDVI corresponded with senescence in forage plants and declining forage availability [27].

Autumn in Montana brings an influx of archery and rifle hunters into mule deer habitat. We estimated the relative amount of hunting activity over space and time by combining available statistics on deer- and elk-hunter effort within our study areas. Every 2 years, MFWP personnel estimated deer (or elk on alternate years) hunter effort by conducting phone surveys [28]. This yielded estimates of the number of hunters and hunter days in each hunting district pursuing elk and deer, separately for archery and rifle hunters and summarized for the entire hunting season. To distribute hunter day totals across daily and weekly time sequences we used independent sets of hunter effort data collected at check stations and through mail surveys. Mandatory hunter check stations were in proximity to each study area where all hunters are required to stop and report what districts were hunted. Check station data were available to estimate relative weekly variation in hunter effort according to hunter-days registered during weekends of the rifle season in the Cabinet-Salish and Whitefish Range study areas and during both weekdays and weekends of rifle season in the Rocky Mountain Front. Lastly, to distribute relative effort across all days of the week where such data were not available from check stations, we used mail survey data collected from moose hunters (N. DeCesare, unpublished data) which included daily hunter effort across every day of the season. We assumed relative variation in hunting effort across days of the week for moose hunters were proportional to those for deer and elk hunters across days of the week and weeks of the season. Such data for moose hunters were collected through voluntary mail surveys sent to all moose license-holders that achieved a 44% response rate (N. DeCesare, unpublished data). Similar to climatic and NDVI variables we also calculated 2-day and weekly averages and subtracted the weekly average from the daily value to capture the daily change in conditions. We also included a categorical hunting season variable to indicate when hunting seasons occurred (non-hunting vs. archery vs. rifle). Licensed hunting of mule deer across the majority of hunting districts in these study areas was focused primarily on antlered male mule deer, with antlerless hunting in only a subset of districts in the Rocky Mountain Front study area (HDs 422, 442, 444, 445). Given this and that hunter effort dedicated towards white-tailed deer and elk was also included in our estimates, our treatment of hunting activity in this analysis is aimed at the general disturbance experienced by animals with the influx of hunters on wildlands during hunting season as opposed to disturbance by hunters specifically targeting the species and sex-class (adult female mule deer) being monitored.

### Statistical analyses

We used a survival, or time-to-event, analysis framework to assess the impacts of the environmental covariates on the initiation of autumn migrations. Most survival analyses assume continuous measurement of time, whereas our data were formatted in discrete days and included many instances of ties in migration timing, wherein multiple animals migrated on the same day. Thus, we employed discrete-time survival analysis models in software R using generalized linear mixed models (GLMM) with a complimentary log-log link function [29]. These GLMM models are comparable to Cox proportional hazards models with log-normal frailty terms but specifically include one or more variables that account for the effect of time itself on the probability of an event occurring. The coefficient(s) for this parameterization of time effectively establishes the baseline hazard function akin to that in continuous-time proportional hazards models [29]. Thus, we first evaluated multiple formulations of an initial base model describing the probability of migration as a function of only time, according to the day since August 26 (referred to as day to migration from here forward). We evaluated multiple treatments of time and mixed effect structures. Specifically, we tested linear, quadratic and log transformed values of time and tested mixed effects for individual deer, year and study area. We treated individual deer-years as independent samples and included random intercepts for each deer in the study and random slopes for the day to migration term.

We employed an information-theoretic model selection approach [30] by first evaluating small suites of candidate models according to each of our five general hypothesizes before subsequently evaluating support across multiple hypotheses with multivariable models [31]. We first grouped our individual and landscape variables into five classes and explored within-class combinations of variables. Variable classes included precipitation (precipitation and SWE), temperature, NDVI, hunting activity, and individual migration characteristics. We conducted AIC-based model selection within each class of variables and carried forward variables that were included in best supported models for each class. We then conducted final modelling across classes by combining these variables into a global model and applying a second stage of model selection. Following Arnold 2010 [32], we discounted models with uninformative parameters where new variable additions did not improve AIC scores by ≥ 2. After AIC model selection we used receiver operator characteristic curves (ROC) and the likelihood ratio chi-squared tests to assess goodness of fit and predictive performance. All continuous variables were scaled and centered prior to statistical analysis, and we checked for collinearity among variables using Pearson correlation coefficients and removed variables from models when correlation coefficients were > 0.6 [33].

## Results

After screening procedures, we included 102 autumn migrations (34 in Cabinet-Fisher, 40 in Rocky Mountain Front, and 28 in Whitefish) from 68 individuals (23 in Cabinet-Fisher, 26 in Rocky Mountain Front, and 19 in Whitefish). The mean day of initiating autumn migrations across all mule deer was October 19 (SD:22 days, range: September 17– December 29), and there was no significant difference between study areas. Mule deer travelled an average of 26.3 km during autumn migration (SD: 11.5 km, 3.9–58.2 km).

The collective annual space use of deer in each study area spanned multiple (up to 9) hunting districts, and individual deer used an average of 2.1, 2.8, and 2.0 hunting districts per year (range 1–4) in the Cabinet-Fisher, Rocky Mountain Front, and Whitefish study areas, respectively. Furthermore, the return of deer to winter ranges occurred over a 3-month time period spanning archery, rifle and closed hunting periods (Fig. 2). While some deer returned relatively early during archery season in September, others remained in summer range into December, after the general rifle season concluded (Fig. 2).

### Initiation of autumn migration

**Fig. 3:**
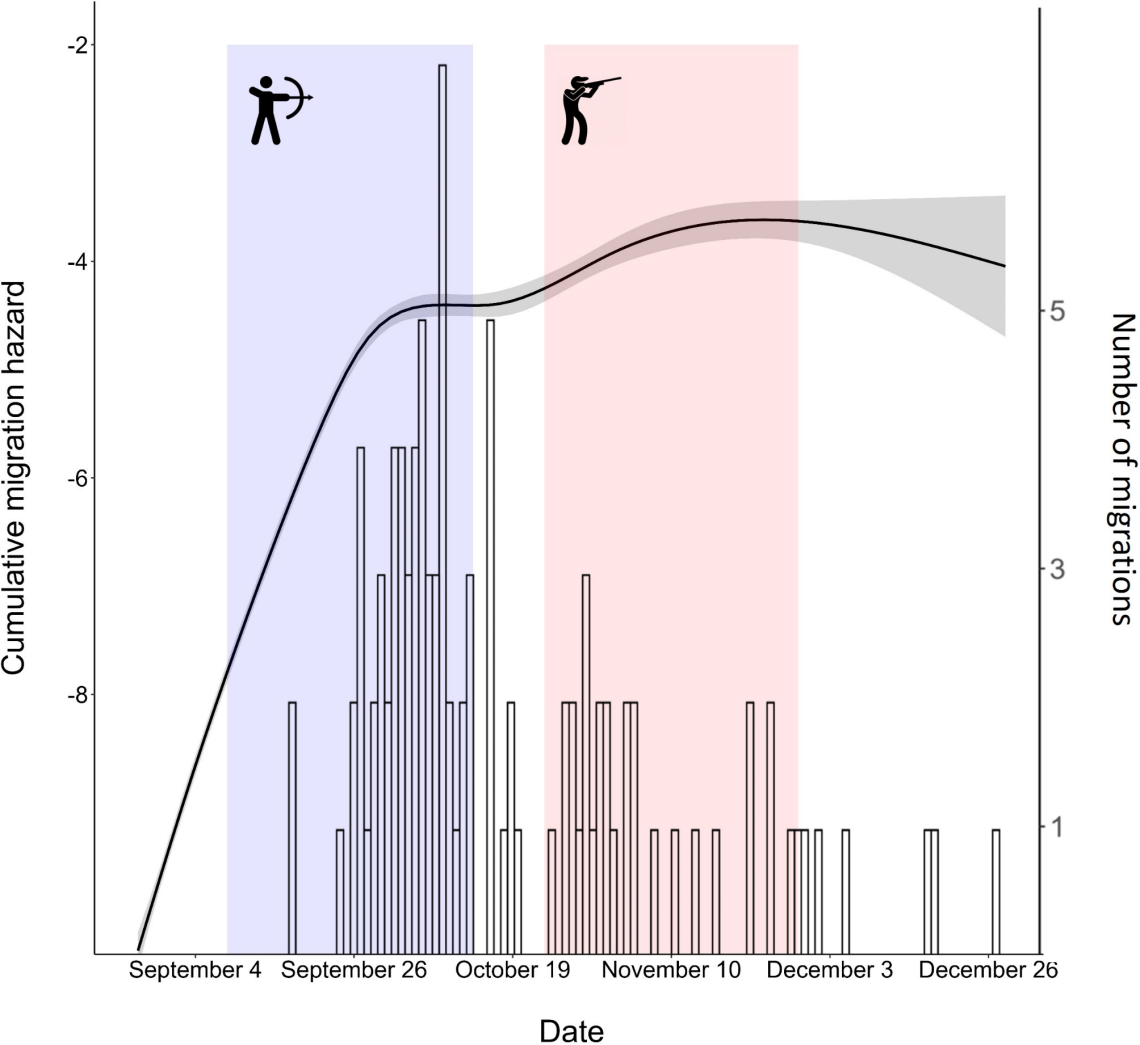
Estimated hazard function (black line) for the initiation of autumn migration for mule deer in 3 study areas of western Montana from discrete-time survival models, 2017–2020. Most migrations, daily counts shown with black histogram, were initiated in late September and early October during the archery hunting season (purple shaded area) and prior to the beginning of the rifle hunt season (red shaded area)

The most parsimonious base model included both a linear and polynomial term for the effect of time on migration probability and included random intercepts for each deer in the study. The random intercept for individual deer was significant with a variance of 3.096 days. Specifically, model predicted values demonstrated that mule deer migration probability began to steeply increase in late September and early October before levelling out in late November and December (Fig. 3).

Adding environmental covariates to our base model for migration initiation showed support for all classes of covariates, including those related to precipitation, temperature, forage, hunting activity, and migration distance. For climatic variables, there were seven precipitation-focused variables with support (within 2 AIC of top model within class), including variables for daily precipitation, daily SWE, 2-day average SWE, 2-day average precipitation, weekly precipitation average, and the difference between daily SWE and the average of the previous week, though weekly precipitation was the top model (Table 1). A model including daily minimum temperature was the top model within the temperature subclass (Table 1). The top model for plant phenology included a term describing the relative difference between current daily NDVI and the end of the season NDVI value (NDVI_senescence_), such that migration probability increased as the daily NDVI values approached that of the NDVI value at the end of the growing season (Table 1). We also tested several temporal variations of hunting activity and the top model included unexpected negative effects of daily or 1-day-lagged estimates of hunting activity (hunting activity_1 − day_) on migration initiation (Table 1). The final model subclass for individual migration characteristics included variables describing the linear distance of migration and the time spent migrating (in days). The top model within this class including only linear distance.

**Table 1:** Model selection results for 5 classes of variables comparing different metrics of precipitation, temperature, forage senescence, hunting activity, and migration distance as they relate to the initiation of autumn migration for mule deer in 3 study areas of western Montana, 2017–2020

Class	Model	df	logLik	AICc	ΔAICc
*Precipitation*					
	Precipitation_week_	5	-415.32	840.65	0.000
	Precipitation_1 − day_ + SWE_change_	6	-414.36	840.74	0.088
	Precipitation_1 − day_	5	-415.52	841.05	0.403
	Precipitation_2 − day_ + SWE_change_	6	-414.55	841.13	0.474
	SWE_change_	5	-415.67	841.36	0.709
	Precipitation_2 − day_	5	-415.68	841.36	0.714
	SWE_2 − day_	5	-416.04	842.10	1.449
	Precipitation_change_	5	-416.77	843.55	2.898
	SWE_1 − day_	5	-417.01	844.04	3.385
	SWE_week_	5	-417.06	844.14	3.486
*Temperature*					
	Temperature_1 − day_	5	-414.19	838.39	0.000
	Temperature_change_	5	-415.67	841.34	2.950
	Temperature_week_	5	-416.51	843.03	4.636
	Temperature_2 − day_	5	-417.07	844.16	5.760
*Forage senescence*					
	NDVI_senescence_	5	-410.74	831.49	0.000
	NDVI_change_	5	-411.85	833.72	2.237
	NDVI_1 − day_	5	-415.81	841.64	10.155
	NDVI_Julian day_	5	-416.46	842.92	11.438
*Hunting activity*					
	Hunting activity_1 − day_	5	-411.51	833.03	0.000
	Hunting activity_change_	5	-414.11	838.23	5.193
	Hunting activity_week_	5	-416.79	843.59	10.554
	Hunting season	6	-417.05	844.11	11.081
*Migration distance*					
	Migration distance	5	-408.81	827.64	0.000
	Migration duration	5	-415.52	841.06	13.419
*Null model (time-only)*		4	-417.08	842.17	0.000

**Fig. 4:**
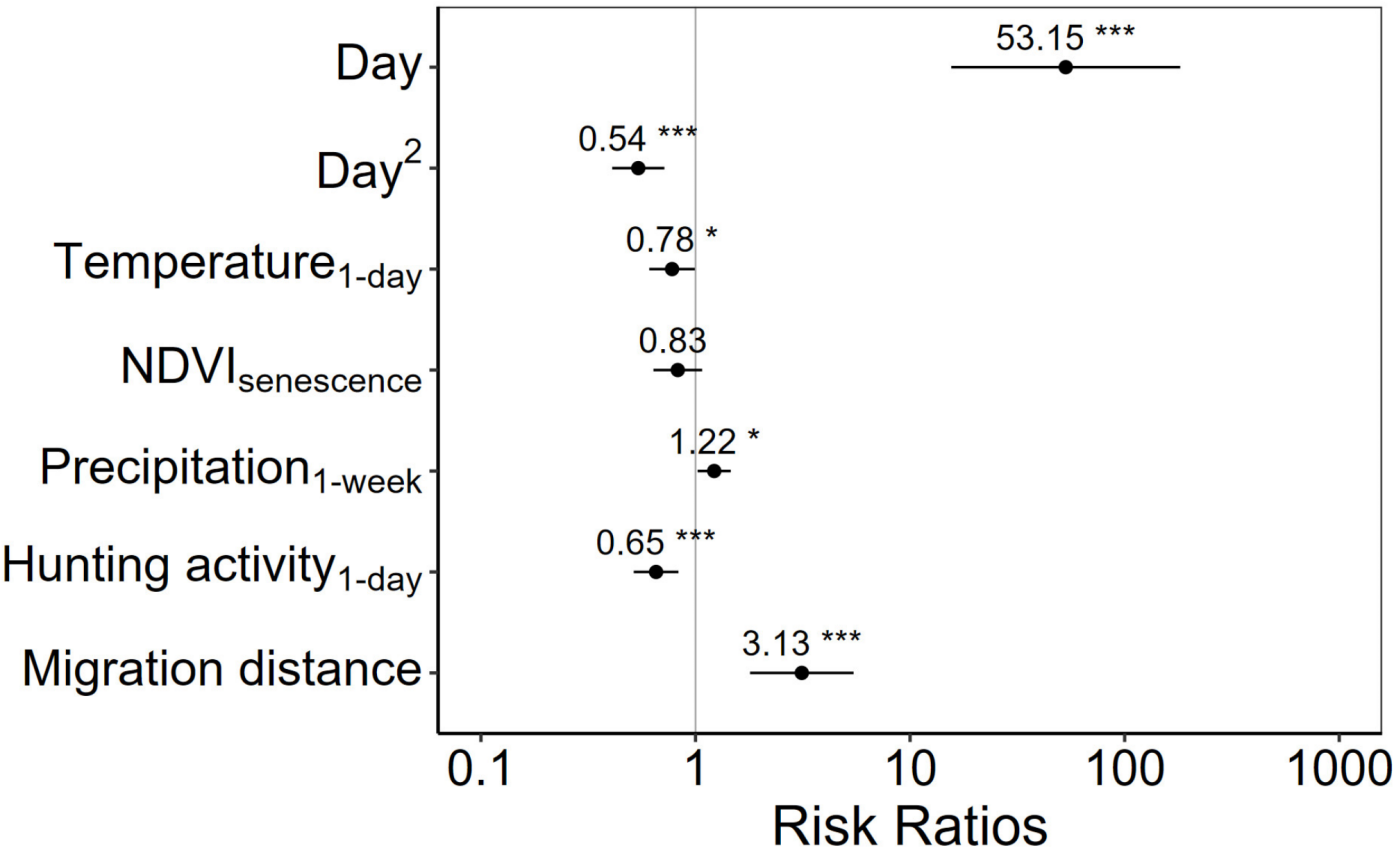
Risk ratio estimates for variables included in the top overall model using discrete-time survival models. Asterisks indicate the level of significance for each variable

When combining variables across all classes, the best model included variables quantifying the daily minimum temperature, weekly precipitation, the distance travelled during migration, the end of season NDVI, daily hunting activity and the underlying curvilinear effect of time (Table 2; Fig. 4). Deer were more likely to migrate when daily minimum temperature decreased (β = -0.252, SE = 0.12, *P* = 0.044) and weekly precipitation increased (β = 0.20, SE = 0.09, *P* = 0.027). Probability of migration increased as migration distance increased (β = 1.141, SE = 0.284, *P* < 0.001), when hunting activity was lower (β = -0.423, SE = 0.122, *P* = 0.0005), and as NDVI fell below the designated end of season NDVI value (β = -0.19, SE = 0.133, *P* = 0.154). The top model demonstrated excellent predictive power (pseudo- *R*^*2*^ = 0.71) and discrimination (ROC = 0.94) and was supported by the likelihood ratio $\:{\chi\:}_{\left(3\right)}^{2}$ = 42.298, *P* < 0.0001.

**Table 2:** Model selection table using AICc for full model set after combining variables from sub class model groupings

Model	df	LogLik	AICc	ΔAICc
Migration Distance + Temperature_1 − day_ + NDVI_senescence_ + Precipitation_1 − week_ + Hunting Activity_1 − day_	9	-390.76	799.55	0
Migration Distance + Hunting Activity_1 − day_	6	-396.83	805.68	6.12
Migration Distance + Temperature_1 − day_ + NDVI_senescence_ + Precipitation_1 − week_	8	-397.70	811.44	11.89
Migration Distance + Temperature_1 − day_ + NDVI_senescence_	7	-399.13	812.28	12.72
Migration Distance + Temperature_1 − day_	6	-400.46	812.94	13.38
Migration Distance + Precipitation_1 − week_	6	-401.53	815.08	15.53
Migration Distance + NDVI_senescence_	6	-402.22	816.46	16.90
Migration Distance	5	-403.40	816.80	17.25

## Discussion

Applying discrete-time survival analyses to our data revealed several key relationships guiding the timing of mule deer autumn migration. First, our results supported the general importance of time (i.e., photoperiod), with migrations occurring at varying probabilistic rates across a 4-month span from September to December. Our polynomial treatment of time was supported over more discrete and categorical effects of archery and rifle hunting seasons, which also occurred within the study period. After accounting for the effect of time, the probability of migration for individual deer were further affected by a number of other spatiotemporal factors. Our results demonstrate that plunging temperatures measured at a daily time scale provided a relatively instantaneous environmental cue for mule deer to begin their migration back to winter range. Precipitation also provided a significant cue for migration, but with less evidence of an instantaneous, daily effect (Table 1). In northwest Montana temperatures often drop below freezing prior to any significant snow accumulation, offering deer an early cue of winter’s onset (Figures S1-S6). As a result, animals may elect to navigate their lengthy migration routes in more direct association with cold temperatures, to proactively avoid energetically taxing deep snow conditions that soon follow in higher elevation summer ranges [5, 7, 11, 34]. Moreover, deer are interpreting changing environmental conditions while considering the distance they must travel during their imminent migration back to winter range and leaving their summer range earlier if their migration route requires longer travel.

While a plant phenology metric (NDVI_senescence_) was retained in the top model, it demonstrated a relatively modest relationship between the probability of migration and plant phenological state (Fig. 4). Specifically, mule deer were more likely to migrate when NDVI values approached the threshold indicating the end of the season and signifying forage conditions had bottomed out at senescent levels, a proxy for plant nutritional value. While ultimate drivers of ungulate migration can include access to improved forage availability [35], our results suggest forage to be a relatively modest factor among others driving the timing at which animals initiate their return. Further research may be worthwhile to evaluate whether this weaker role is due to the parallel deterioration of forage resources on winter ranges, such that the discrepancy between conditions in summer and winter ranges in autumn is more strongly a function of weather than of forage conditions [36].

Though harvest of adult female mule deer was either closed (Cabinet-Fisher and Whitefish Range) or limited (Rocky Mountain Front) within our study system, the added presence of hunters seeking elk, white-tailed deer, and male mule deer on the landscape can affect female mule deer movement even when they are not being targeted [37]. Our results relating to hunting activity ran counter to our a priori hypothesis and showed deer migrating during periods of lower hunter activity (Fig. 4). Timing of autumn migration peaked in late September and early October, aligning with times of lower hunting activity prior to the general rifle season (Fig. 3). Thus, instead of high hunting activity serving as a trigger to leave summer range as hypothesized [*sensu*^8^], deer were statistically more likely to initiate migrations before hunting activity increased to its peak during rifle season. Possible interpretations of this result include early migrations in anticipation of forthcoming higher risk either along migration paths or within summer range. Hunting activity likely varies in response to patterns of land ownership, road access, and terrain heterogeneity at finer spatial scales than we were able to measure here, and thus we were unable to further evaluate these possibilities. Perhaps, deer migrated early to minimize exposure to hunting risk on winter range private lands in some situations [e.g., 38] and that deer faced higher risk during the migration period, which is itself inherently risky [39–41]. Combining this with changing environmental conditions in autumn, mule deer may seek to undergo migration earlier to avoid cumulative adversity [8, 41]. Moreover, a mule deer’s body condition, associated with age and fat reserves, could also play a role in triggering autumn migration, but this data was not available for this study [42].

Our results highlight a challenge for wildlife managers where the spatial scales over which ecological processes influencing populations occur, such as migration distances, often exceed the scales at which populations are managed. We found that mule deer traversed the boundaries of multiple hunting districts during the hunting season, exposing certain sub-groups of deer to different harvest regimes within this period. For example, on the Rocky Mountain Front, several deer spent summers in hunting district 150, an area where only antlered harvest was permitted and mule deer populations were below management objectives (with a record-low harvest 3 bucks, 5% of the long-term average, occurring in 2018) [19]. In autumn, many of these deer migrated to hunting district 425 (Fig. 1), where harvest of females was permitted, potentially hampering population growth in hunting district 150 depending upon the timing of those autumn migrations. In this case, the longer migration distances to reach summer range in HD150 would dictate generally earlier autumn migrations and higher exposure to antlerless hunting on winter range. This anecdote serves to illustrate a broader phenomenon, whereby management actions in certain hunting districts could be counter to population objectives of nearby districts as dictated by the timing of autumn migrations.

## Conclusions

Intensifying autumn weather patterns provide the clearest motivation for mule deer migration initiation [7, 43–46]. Nonetheless, individual variation in migration timing within populations suggest animal-specific characteristics like proximity of summer home range to wintering grounds can play concomitant roles in migration behavior [43]. This variation in migratory timing during the autumn hunting season led to variable exposure to human hunters as deer traversed multiple management boundaries. Knowing where and when a group of animals will be is an essential component for developing area-specific management scenarios that effectively address unequal harvest vulnerabilities among individuals while maximizing hunter opportunities temporally and spatially.

## Electronic supplementary material

Below is the link to the electronic supplementary material.

Supplementary Material 1

## Data Availability

The data that support the findings of this study are available from the corresponding author upon reasonable request.

## Abbreviations

GLMM: Generalized linear mixed model
GPS: Global positioning system
Hunting District: HD
NDVI: Normalized difference vegetation index
ROC: Receiver operator characteristic
SWE: Snow water equivalence
