# Supplementary material for: Distance, weather, and forage conditions drive timing of autumn migration in female mule deer
Source: Mov Ecol. 2025 Feb 25;13:10. doi: 10.1186/s40462-025-00540-x (PMC11863777; doi:10.1186/s40462-025-00540-x)

Distance, weather and forage conditions drive timing of autumn migration in female mule deer

Colby B. Anton*, Montana Cooperative Wildlife Research Unit, University of Montana, Missoula, MT 59812, USA

Nicholas J. DeCesare, Montana Fish, Wildlife and Parks, Missoula, MT 59804, USA

Collin J. Peterson, Montana Fish, Wildlife and Parks, Kalispell, MT 59901, USA

*Current Affiliation: Montana Fish, Wildlife and Parks, Missoula, MT 59804, USA

| Table S1. AICc model selection table for the precipitation and snow water equivalence (SWE) subclass of variable modeling. All models included time variables. | | | | |
| --- | --- | --- | --- | --- |
| Model | df | logLik | AICc | ΔAICc |
| 1 week cumulative precipitation | 5 | -415.32 | 840.65 | 0.000 |
| 1 week difference SWE + 1 week cumulative precipitation | 6 | -414.32 | 840.66 | 0.009 |
| 1 week difference SWE + 1 week average precipitation | 6 | -414.32 | 840.66 | 0.009 |
| 1 week difference SWE + daily precipitation | 6 | -414.36 | 840.74 | 0.088 |
| daily precipitation | 5 | -415.52 | 841.05 | 0.403 |
| 1 week difference SWE + 2 day average precipitation | 6 | -414.55 | 841.13 | 0.474 |
| 1 week difference SWE | 5 | -415.67 | 841.36 | 0.709 |
| 2 day average precipitation | 5 | -415.68 | 841.36 | 0.714 |
| 2 day average SWE | 5 | -416.04 | 842.10 | 1.449 |
| Daily SWE + 1 week cumulative precipitation | 6 | -415.24 | 842.49 | 1.843 |
| Daily SWE + 1 week average precipitation | 6 | -415.24 | 842.49 | 1.843 |
| 1 week difference SWE + 1 week difference precipitation | 6 | -415.38 | 842.79 | 2.134 |
| Daily SWE + daily precipitation | 6 | -415.47 | 842.95 | 2.303 |
| Daily SWE + 2 day average precipitation | 6 | -415.60 | 843.23 | 2.577 |
| 1 week difference precipitation | 5 | -416.77 | 843.55 | 2.898 |
| Daily SWE | 5 | -417.01 | 844.04 | 3.385 |
| 1 week cumulative SWE | 5 | -417.06 | 844.14 | 3.486 |
| 1 week average SWE | 5 | -417.06 | 844.14 | 3.486 |
| SWE + 1 week difference precipitation | 6 | -416.71 | 845.44 | 4.784 |

| Table S2. AICc model selection table for the minimum temperature subclass of variable modeling. All models included time variables. | | | | |
| --- | --- | --- | --- | --- |
| Model | df | logLik | AICc | ΔAICc |
| Minimum temperature | 5 | -414.19 | 838.39 | 0.000 |
| 1 week difference minimum temperature | 5 | -415.67 | 841.34 | 2.950 |
| 1 week average minimum temperature | 5 | -416.51 | 843.03 | 4.636 |
| 2 day average minimum temperature | 5 | -417.07 | 844.16 | 5.760 |

| Table S3. AICc model selection table for the Normalized Difference Vegetation Index (NDVI) subclass of variable modeling. All models included time variables. | | | | |
| --- | --- | --- | --- | --- |
| Model | df | logLik | AICc | ΔAICc |
| Change in NDVI since end of season | 5 | -410.74 | 831.49 | 0.000 |
| 1 week difference NDVI | 5 | -411.85 | 833.72 | 2.237 |
| Daily NDVI | 5 | -415.81 | 841.64 | 10.155 |
| Days since end of season | 5 | -416.46 | 842.92 | 11.438 |

| Table S4. AICc model selection table for the hunting pressure subclass of variable modeling. All models included time variables. | | | | |
| --- | --- | --- | --- | --- |
| Model | df | logLik | AICc | ΔAICc |
| 1 day lag hunting pressure | 5 | -411.51 | 833.03 | 0.000 |
| Daily hunting pressure | 5 | -411.51 | 833.03 | 0.001 |
| 1 week difference hunting pressure | 5 | -414.11 | 838.23 | 5.194 |
| 1 week average hunting pressure | 5 | -416.79 | 843.59 | 10.555 |
| Hunting season | 6 | -417.05 | 844.11 | 11.082 |

| Table S5. AICc model selection table for the migration distance and duration subclass of variable modeling. All models included time variables. | | | | |
| --- | --- | --- | --- | --- |
| Model | df | logLik | AICc | ΔAICc |
| Linear distance of migration | 5 | -408.81 | 827.64 | 0.000 |
| Duration of migration | 5 | -415.52 | 841.06 | 13.419 |


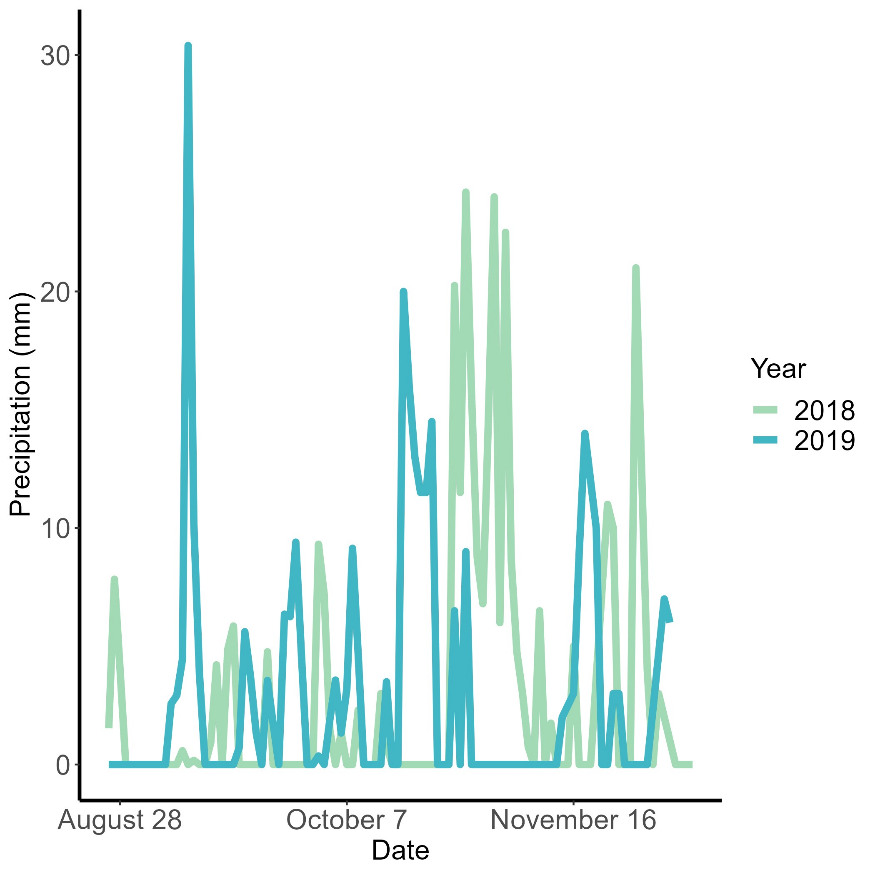
Figure S1. Daily Precipitation for each year included in this study for the Cabinet-Salish study area

Figure S2. Daily Precipitation for each year included in this study for the Rocky Mountain Front study area


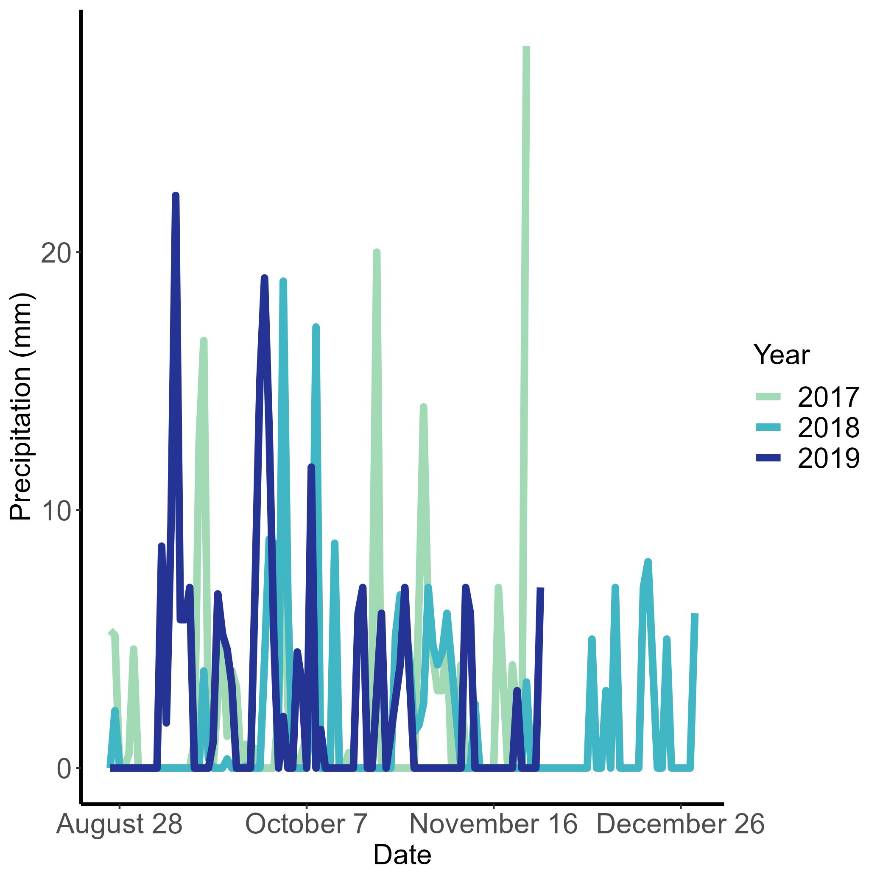


Figure S3. Daily Precipitation for each year included in this study for the Whitefish study area


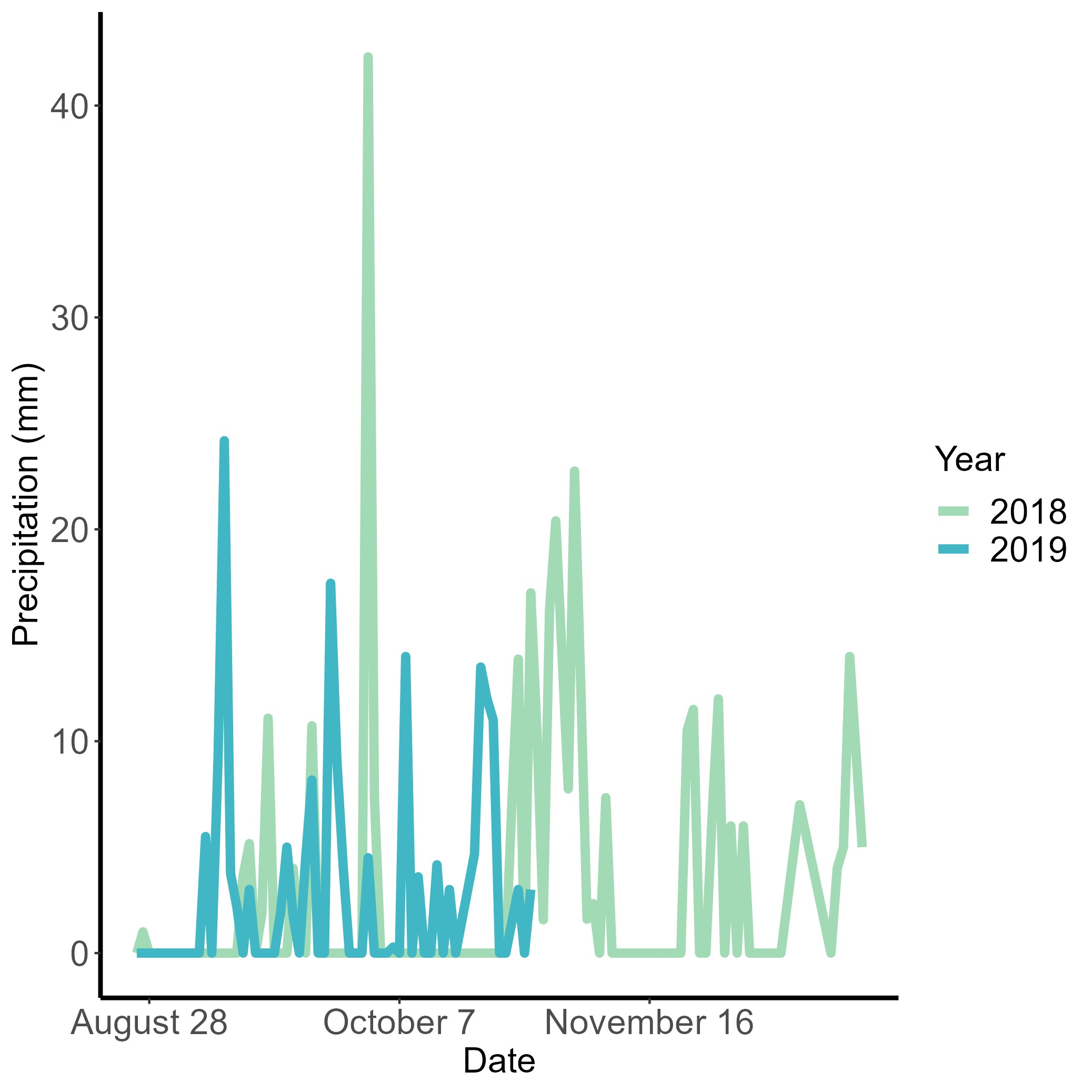


Figure S4. Daily minimum temperature for each year included in this study for the Cabinet-Salish study area.


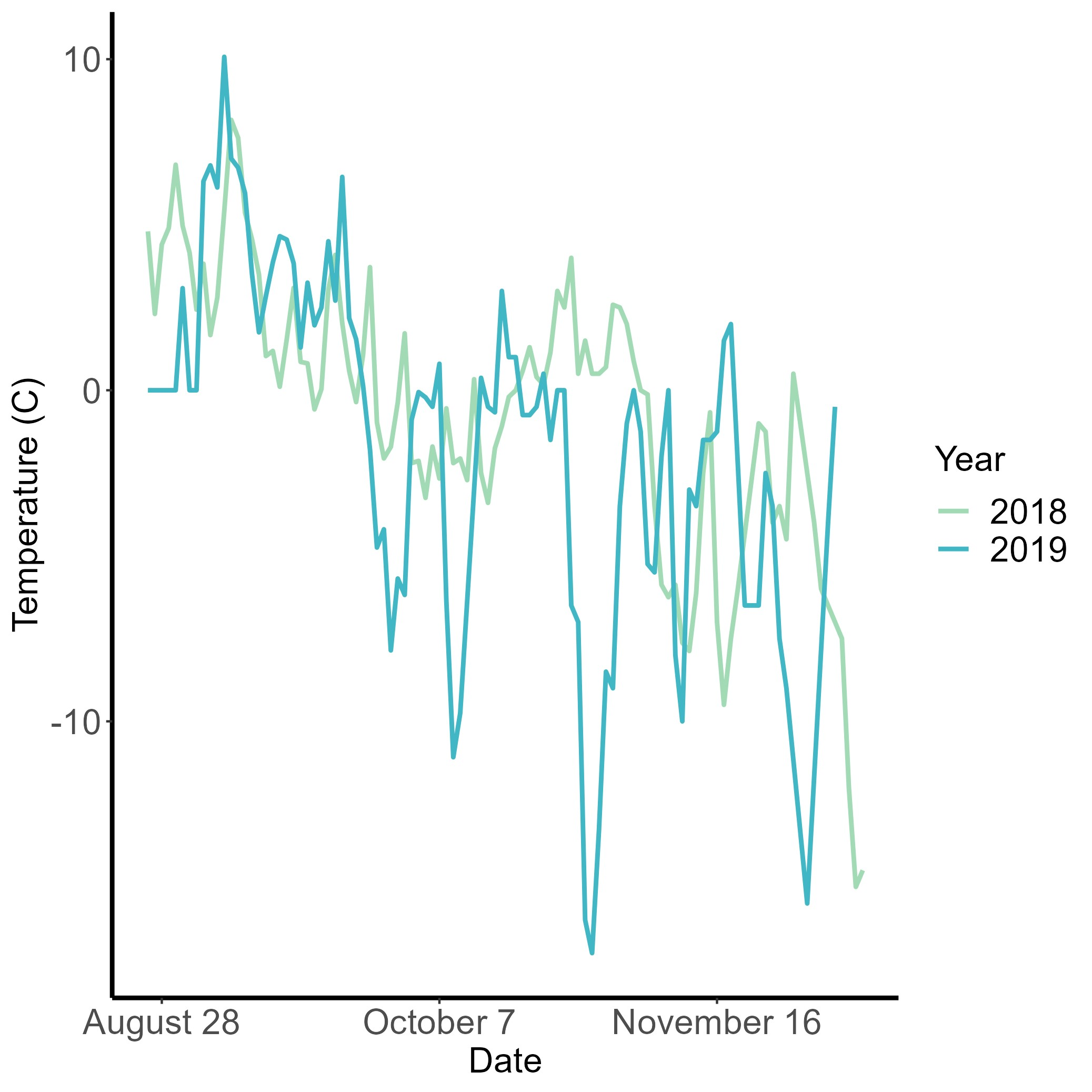


Figure S5. Daily minimum temperature for each year included in this study for the Rocky Mountain Front study area.


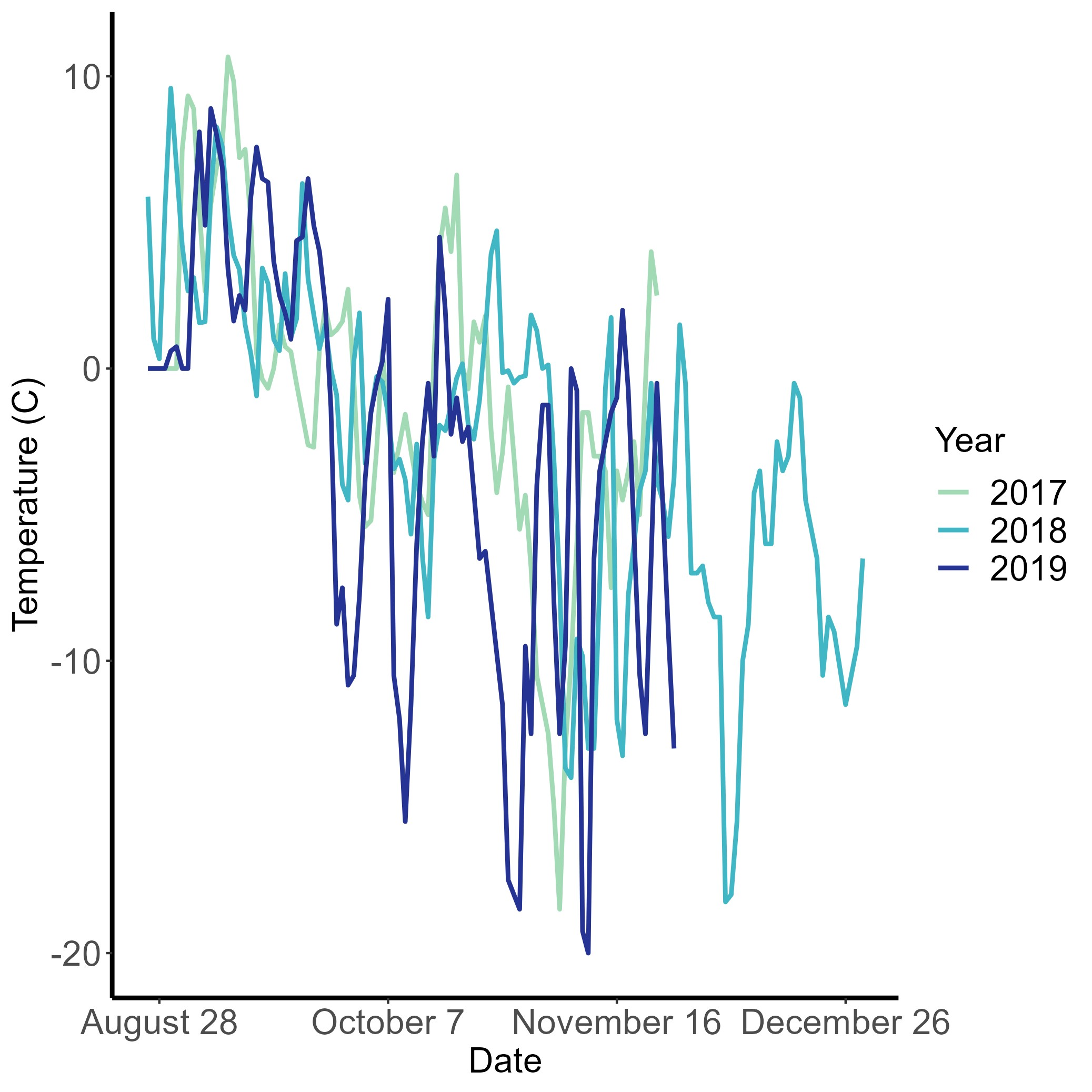


Figure S6. Daily minimum temperature for each year included in this study for the Whitefish study area.


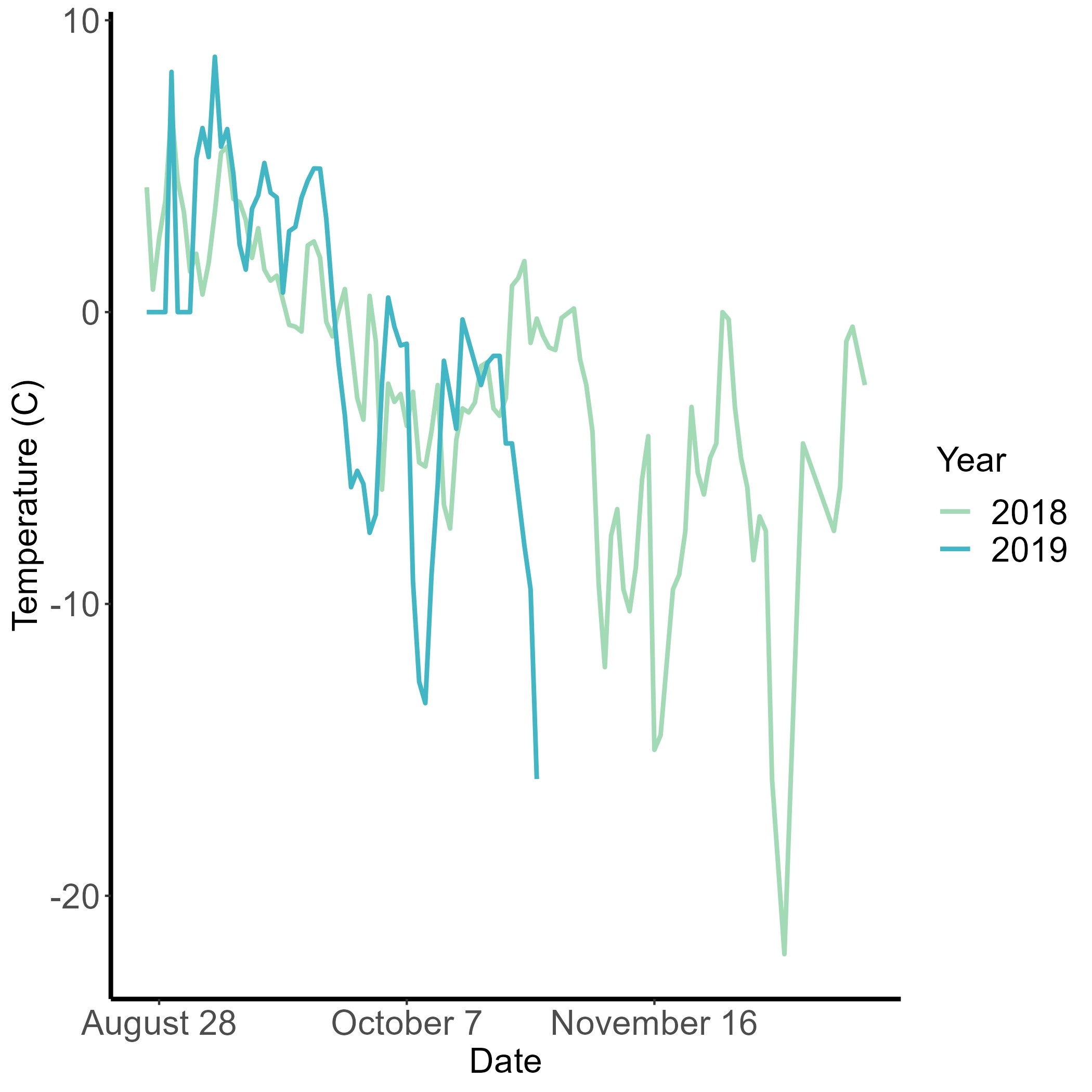


Figure S7. Estimated daily hunting pressure for all three study areas across archery and rifle season (approximately September 1 – November 30 annually) in northwest Montana. Daily estimates were derived from a combination of data from bi-annual hunter phone surveys and moose hunter surveys.


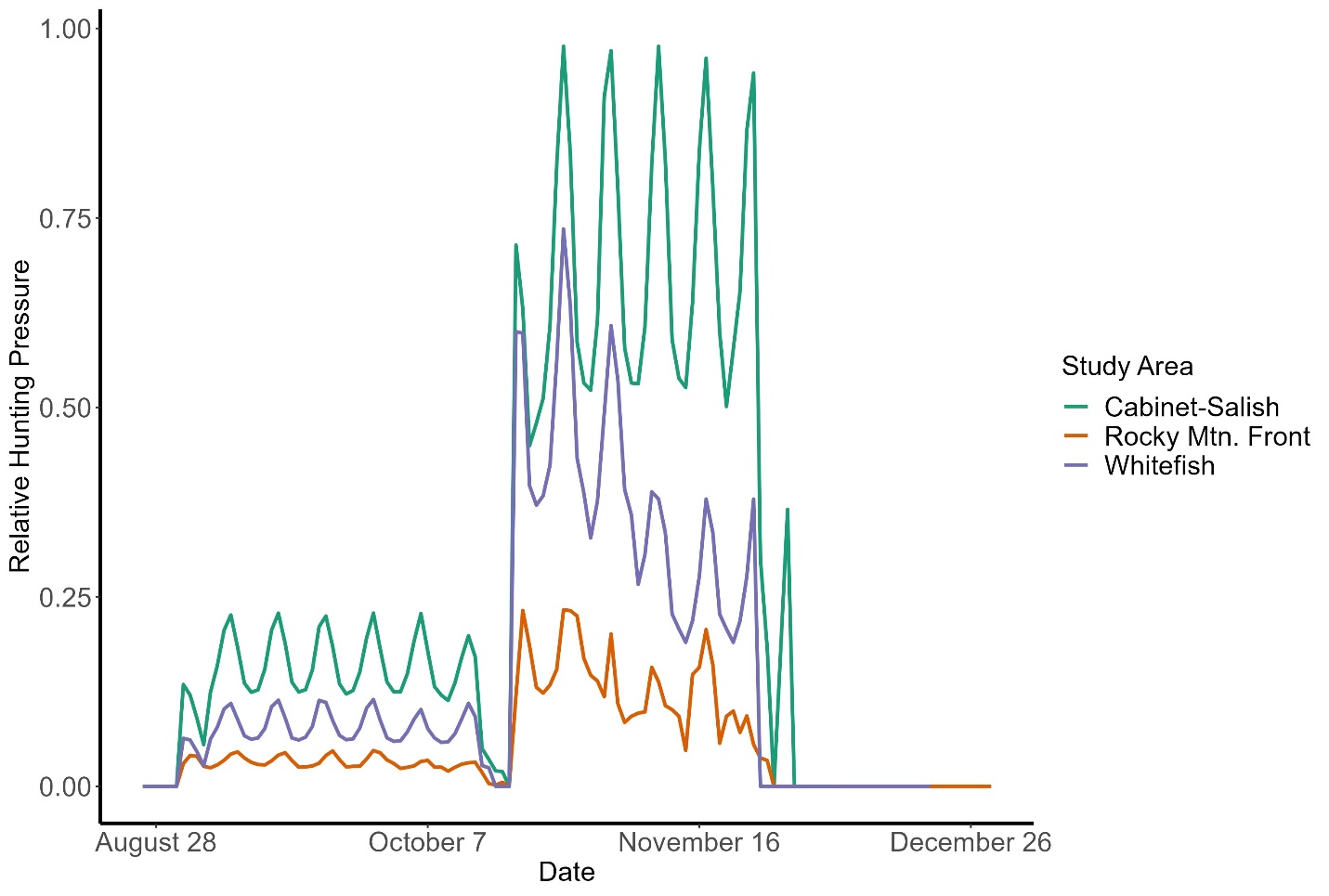

Supplement: Supplementary file 1 — Supplementary Material 1 [file 40462_2025_540_MOESM1_ESM.docx]
